# A Meta-Analysis Approach for Characterizing Pan-Cancer Mechanisms of Drug Sensitivity in Cell Lines

**DOI:** 10.1371/journal.pone.0103050

**Published:** 2014-07-18

**Authors:** Kendric Wang, Raunak Shrestha, Alexander W. Wyatt, Anupama Reddy, Joseph Lehár, Yuzhou Wang, Anna Lapuk, Colin C. Collins

**Affiliations:** 1 Vancouver Prostate Centre, Vancouver General Hospital, Vancouver, Canada; 2 CIHR/MSHFR Bioinformatics Training Program, University of British Columbia, Vancouver, Canada; 3 Department of Urologic Sciences, the University of British Columbia, Vancouver, Canada; 4 Novartis Pharmaceuticals, Oncology Division, Basal, Switzerland; Institute of Clinical Physiology, c/o Toscana Life Sciences Foundation, Italy

## Abstract

Understanding the heterogeneous drug response of cancer patients is essential to precision oncology. Pioneering genomic analyses of individual cancer subtypes have begun to identify key determinants of resistance, including up-regulation of multi-drug resistance (MDR) genes and mutational alterations of drug targets. However, these alterations are sufficient to explain only a minority of the population, and additional mechanisms of drug resistance or sensitivity are required to explain the remaining spectrum of patient responses to ultimately achieve the goal of precision oncology. We hypothesized that a pan-cancer analysis of *in vitro* drug sensitivities across numerous cancer lineages will improve the detection of statistical associations and yield more robust and, importantly, recurrent determinants of response. In this study, we developed a statistical framework based on the meta-analysis of expression profiles to identify pan-cancer markers and mechanisms of drug response. Using the Cancer Cell Line Encyclopaedia (CCLE), a large panel of several hundred cancer cell lines from numerous distinct lineages, we characterized both known and novel mechanisms of response to cytotoxic drugs including inhibitors of Topoisomerase 1 (TOP1; Topotecan, Irinotecan) and targeted therapies including inhibitors of histone deacetylases (HDAC; Panobinostat) and MAP/ERK kinases (MEK; PD-0325901, AZD6244). Notably, our analysis implicated reduced replication and transcriptional rates, as well as deficiency in DNA damage repair genes in resistance to TOP1 inhibitors. The constitutive activation of several signaling pathways including the interferon/STAT-1 pathway was implicated in resistance to the pan-HDAC inhibitor. Finally, a number of dysregulations upstream of MEK were identified as compensatory mechanisms of resistance to the MEK inhibitors. In comparison to alternative pan-cancer analysis strategies, our approach can better elucidate relevant drug response mechanisms. Moreover, the compendium of putative markers and mechanisms identified through our analysis can serve as a foundation for future studies into these drugs.

## Introduction

Over the past decade, cancer treatment has seen a gradual shift towards ‘precision medicine’ and making rational therapeutic decisions for a patient's cancer based on their distinct molecular profile. However, broad adoption of this strategy has been hindered by an incomplete understanding for the determinants that drive tumour response to different cancer drugs. Intrinsic differences in drug sensitivity or resistance have been previously attributed to a number of molecular aberrations. For instance, the constitutive expression of almost four hundred multi-drug resistance (MDR) genes, such as ATP-binding cassette transporters, can confer universal drug resistance in cancer [1]. Similarly, mutations in cancer genes (such as EGFR) that are selectively targeted by small-molecule inhibitors can either enhance or disrupt drug binding and thereby modulate cancer drug response [2]. In spite of these findings, the clinical translation of MDR inhibitors have been complicated by adverse pharmacokinetic interactions [3]. Likewise, the presence of mutations in targeted genes can only explain the response observed in a fraction of the population, which also restricts their clinical utility. As an example of the latter, lung cancers initially sensitive to EGFR inhibition acquire resistance which can be explained by EGFR mutations in only half of the cases. Other molecular events, such as MET proto-oncogene amplifications, have been associated with resistance to EGFR inhibitors in 20% of lung cancers independently of EGFR mutations [4]. Therefore, there is still a need to uncover additional mechanisms that can influence response to cancer treatments.

Historically, gene expression profiling of *in vitro* models have played an essential role in investigating determinants underlying drug response [5]–[8]. Specifically, cell line panels compiled for individual cancer types have helped identify markers predictive of lineage-specific drug responses, such as associating P27(KIP1) with Trastuzumab resistance in breast cancers and linking epithelial-mesenchymal transition genes to resistance to EGFR inhibitors in lung cancers [9]–[11]. However, application of this strategy has been limited to a handful of cancer types (e.g. breast, lung) with sufficient numbers of established cell line models to achieve the statistical power needed for new discoveries.

**Figure 1:**
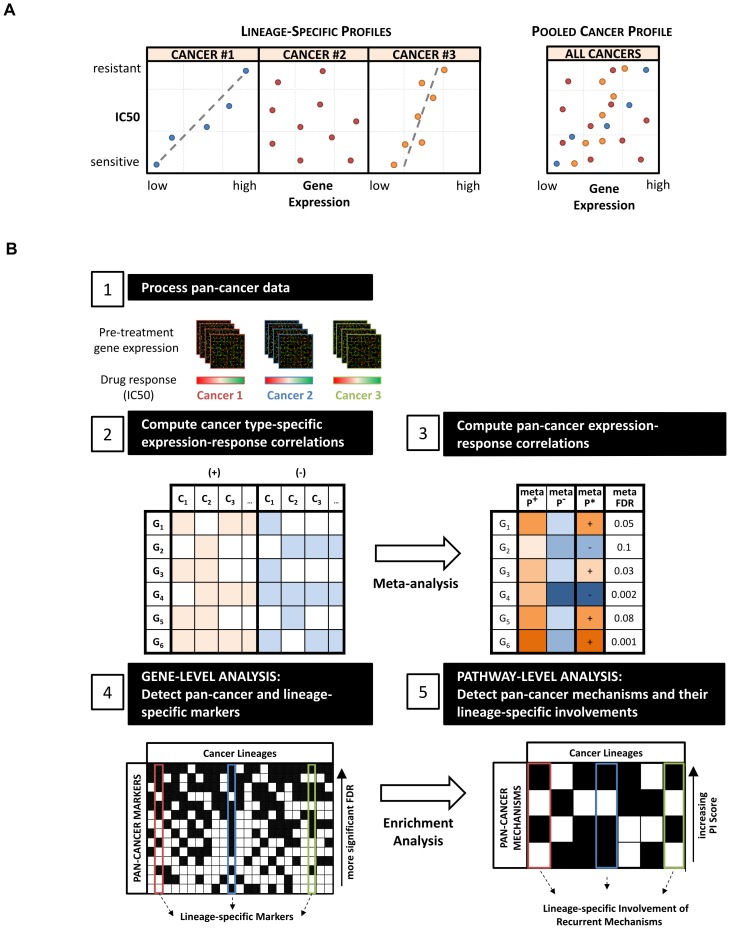
Pan-cancer analysis strategy. (A) Schematic demonstrating a major drawback of the commonly-used pooled cancer approach (PC-Pool), namely that the gene expression and pharmacological profiles of samples from different cancer lineages are often incomparable and therefore inadequate for pooling together into a single analysis. (B) Workflow depicting our PC-Meta approach. First, each cancer lineage in the pan-cancer dataset is independently assessed for gene expression-drug response correlations in both positive and negative directions (Step 2). Then, a meta-analysis method is used to aggregate lineage-specific correlation results and to determine pan-cancer expression-response correlations. The significance of these correlations is indicated by multiple-test corrected p-values (meta-FDR; Step 3). Next, genes that significantly correlate with drug response across multiple cancer lineages are identified as pan-cancer gene markers (meta-FDR <0.01; Step 4). Finally, biological pathways significantly enriched in the discovered set of pan-cancer gene markers are identified as pan-cancer mechanisms of response (PI Score >1.0; Step 5). A subset of the pan-cancer markers correlated with drug response in individual cancer lineages are selected as lineage-specific markers. The involvement levels of pan-cancer mechanisms in individual cancer lineages are calculated from the pathway enrichment analysis of these lineage-specific markers.

Recent studies addressed the problem of limited sample sizes by investigating *in vitro* drug sensitivity in a pan-cancer manner, across large cell line panels that combine multiple cancer types screened for the same drugs [7], [8], [12], [13]. In this way, pan-cancer analysis can improve the testing for statistical associations and help identify dysregulated genes or oncogenic pathways that recurrently promote growth and survival of tumours of diverse origins [14], [15]. The common approach used for pan-cancer analysis directly pools samples from diverse cancer types; however, this has two major disadvantages. First, when samples are considered collectively, significant gene expression-drug response associations present in smaller sized cancer lineages can be obscured by the lack of associations present in larger sized lineages. Second, the range of gene expressions and drug pharmacodynamics values are often lineage-specific and incomparable between different cancer lineages (**Figure 1A**). Collectively, these issues reduce the potential to detect meaningful associations common across multiple cancer lineages.

To tackle the problems introduced through the direct pooling of data, we developed a statistical framework based on meta-analysis called ‘PC-Meta’. PC-Meta identifies pan-cancer markers and mechanisms of drug response by testing for gene expression-drug response associations in each cancer lineage individually and combining the results from each lineage. Prior studies have successfully applied meta-analyses to combine incompatible genomic datasets for a single cancer type, and to combine datasets from different cancers to identify common mechanisms of cancer initiation and progression [16]–[18]. To our knowledge, this is the first study to leverage meta-analysis in the identification of intrinsic pan-cancer determinants of response to cancer therapy.

## Materials and Methods

### Cancer Cell Line Encyclopaedia (CCLE) Dataset

The CCLE pan-cancer dataset used in this study encompasses 1046 cancer cell lines derived from 24 cancer types and screened for pharmacological sensitivity to 24 anti-cancer compounds [8]. The pre-processed gene expression and drug sensitivity data were directly obtained from the CCLE project (http://www.broadinstitute.org/ccle/home; GSE36139). Cell lines were profiled prior to treatment for gene expression using the Affymetrix U133plus2.0 array, and for mutations in 33 known cancer genes by mass spectrometric genotyping (OncoMap). Inhibitory concentration 50 (IC50) values extrapolated in the original study from dose response data were used as the measure of drug effectiveness.

### Meta-analysis Approach to Pan-Cancer Analysis

Our PC-Meta approach for the identification of pan-cancer markers and mechanisms of drug response is illustrated in **Figure 1B**. Initially, each cancer lineage in the pan-cancer dataset was treated as a distinct dataset and independently assessed for associations between baseline gene expression levels and drug response values. These lineage-specific expression-response correlations were calculated using the Spearman's rank correlation test. Lineages that exhibited minimal differential drug sensitivity value (having fewer than three samples or an log_10_(IC50) range of less than 0.5) were excluded from analysis.

Then, results from the individual lineage-specific correlation analyses were combined using meta-analysis to determine pan-cancer expression-response associations. We used Pearson's method [19], a one-tailed Fisher's method for meta-analysis. Fisher's method is a standard technique that aggregates multiple p-values into a single meta P-value where a small meta P-value indicates significant expression-response correlation in one or more cancer lineages. Pearson's method can reduce false associations resulting from conflicting directions of correlation in different lineages. It combines individual lineage p-values for positive and negative correlations separately and returns the more significant of the two combined values (meta P^+^ and meta P^-^) as the final meta P-value (meta P*). From this, a multiple-test corrected meta P-value (meta-FDR) was calculated using the Benjamini-Hochberg (BH) method. For each drug, genes with meta-FDR <0.01 were considered pan-cancer markers of response.

Next, pan-cancer mechanisms of response were revealed by performing pathway enrichment analysis on the discovered pan-cancer markers using the Ingenuity Pathway Analysis software (IPA; Ingenuity Systems, Inc., Redwood City, CA). The statistical over-representation of canonical IPA pathways was calculated using Fischer's exact test and BH multiple-test correction method. A ‘pathway involvement (PI) score’ was calculated for each pathway as the -log_10_(BH-corrected pathway enrichment p-value). Pathways with PI score >1.0 were considered significantly associated with drug response.

Finally, since pan-cancer markers may be relevant in only a subset of cancer lineages, we defined sets of genes associated with response in each lineage as lineage-specific markers. Lineage-specific markers were derived as the subset of pan-cancer markers that significantly correlated with response in a given lineage (Spearman's rank correlation test p-value <0.05 and |Spearman's correlation coefficient| >0.3). Since pan-cancer mechanisms may similarly be involved in only a subset of cancer lineages, their involvement in each lineage was delineated through the pathway enrichment analysis of lineage-specific gene markers as described above.

### Alternative Approaches to Pan-Cancer Analysis

We evaluated PC-Meta against two alternative approaches commonly used in prior studies for identifying pan-cancer markers and mechanisms. One of them, which we termed ‘PC-Pool’, identifies pan-cancer markers as genes that correlate with drug response in a pooled dataset of multiple cancer lineages [8], [12]. Statistical significance was determined based on the same statistical test of Spearman's rank correlation with BH multiple test correction (BH-corrected p-values <0.01 and |Spearman's rho, r_s_|>0.3). Pan-cancer mechanisms were revealed by performing pathway enrichment analysis on these pan-cancer markers.

A second alternative approach, which we termed ‘PC-Union’, naively identifies pan-cancer markers as the union of response-associated genes detected in each cancer lineage [20]. Response-associated markers in each lineage were also identified using the Spearman's rank correlation test with BH multiple test correction (BH-corrected p-values <0.01 and |r_s_|>0.3). Pan-cancer mechanisms were revealed by performing pathway enrichment analysis on the collective set of response-associated markers identified in all lineages.

## Results and Discussion

### Strategy for Pan-Cancer Analysis

We developed PC-Meta, a two stage pan-cancer analysis strategy, to investigate the molecular determinants of drug response (**Figure 1B**). Briefly, in the first stage, PC-Meta assesses correlations between gene expression levels with drug response values in all cancer lineages independently and combines the results in a statistical manner. A meta-FDR value calculated for each gene is used to pinpoint genes that are recurrently associated with response in multiple cancer types and therefore are potential pan-cancer markers. In the second stage, the pan-cancer gene markers are mapped to cell signaling pathways to elucidate pan-cancer mechanisms involved in drug response. To test our approach, we applied PC-Meta to the CCLE dataset, a large pan-cancer cell line panel that has been extensively screened for pharmacological sensitivity to numerous cancer drugs. PC-Meta was evaluated against two commonly used pan-cancer analysis strategies, which we termed ‘PC-Pool’ and ‘PC-Union’. PC-Pool identifies pan-cancer markers as genes that are associated with drug response in a pooled dataset of cancer lineages. PC-Union, a simplistic approach to meta-analysis (not based on statistical measures), identifies pan-cancer markers as the union of response-correlated genes detected in each cancer lineage. Additional details of PC-Meta, PC-Pool, and PC-Union are provided in the Methods section.

### Selecting CCLE Compounds Suitable for Pan-Cancer Analysis

**Figure 2:**
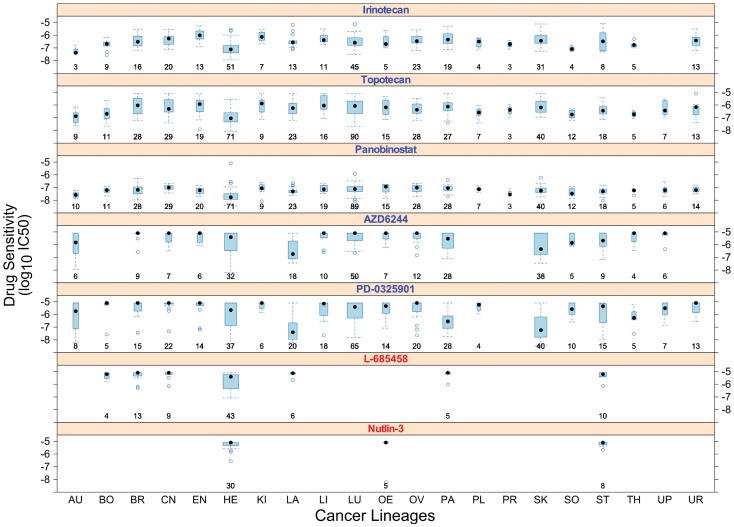
Drug response across different cancer lineages for a subset of CCLE compounds. Boxplots indicate the distribution of drug sensitivity values (based on IC50) in each cancer lineage to each cancer drug. For example, most cancer lineages are resistant to L-685458 (with IC50 around 10^−5^ M) except for haematopoietic cancers (IC50 from 10^−5^ to 10^−8^ M). The number of samples in a cancer lineage screened for drug response is shown under the corresponding boxplot. Compounds denoted in blue text exhibited a broad range of responses in multiple cancer lineages and were selected for analysis in this study, whereas compounds denoted in red text are examples of compounds excluded from analysis. Cancer lineage abbreviations – **AU**: autonomic; **BO**: bone; **BR**: breast; **CN**: central nervous system; **EN**: endometrial; **HE**: haematopoietic/lymphoid; **KI**: kidney; **LA**: large intestine; **LI**: liver; **LU**: lung; **OE**: oesophagus; **OV**: ovary; **PA**: pancreas; **PL**: pleura; **SK**: skin; **SO**: soft tissue; **ST**: stomach; **TH**: thyroid; **UP**: upper digestive; **UR**: urinary

24 compounds available from the CCLE resource were evaluated to determine their suitability for pan-cancer analysis. For eight compounds, none of the pan-cancer analysis methods returned sufficient markers (more than 10 genes) for follow-up and were therefore excluded from subsequent analysis (**Table S1**). Failure to identify markers for these drugs can be attributed to either an incomplete compound screening (i.e. performed on a small number of cancer lineages) such as with Nutlin-3, or the cancer type specificity of compounds such as with Erlotinib, which is most effective in EGFR-addicted non-small cell lung cancers (**Figure S1**). Seven additional compounds, including L-685458 and Sorafenib, exhibited dynamic response phenotypes in only one or two lineages and were also considered inappropriate for pan-cancer analysis (**Figure 2** **; Figure S1**). Even though the PC-Pool strategy identified numerous gene markers associated with response to these seven compounds, close inspection of these markers indicated that many of them actually corresponded to molecular differences between lineages rather than relevant determinants of drug response. For instance, L-685458, an inhibitor of AβPP γ-secretase activity, displayed variable sensitivity in hematopoietic cancer cell lines and primarily resistance in all other cancer lineages. As a result, the identified 815 gene markers were predominantly enriched for biological functions related to Hematopoetic System Development and Immune Response (**Table S2**). This highlights the limitations of directly pooling data from distinct cancer lineages. Out of the remaining nine compounds, we focused on five drugs that belonged to distinct classes of inhibitors (targeting TOP1, HDAC, and MEK) and exhibited a broad range of responses in multiple cancer lineages (**Figure 2** **,** **Table 1**).

**Table 1:** Number of gene markers significantly correlated with response to different drugs identified by PC-Meta, PC-Pool, and PC-Union approaches.

Compound	Target(s)	No. of PC-Meta Markers	No. of PC-Pool Markers (Overlap with PC-Meta)	No. of PC-Union Markers (Overlap with PC-Meta)
**Irinotecan**	TOP1	211	832 (105; 13%)	30 (19; 63%)
**Topotecan**	TOP1	757	474 (256; 54%)	61 (57; 93%)
**Panobinostat**	HDAC	542	723 (200; 28%)	58 (46; 79%)
**AZD6244**	MEK	10	51 (6; 12%)	7 (1; 14%)
**PD-0325901**	MEK	171	46 (23; 50%)	156 (29; 19%)

### Intrinsic Determinants of Response to TOP1 Inhibitors (Topotecan and Irinotecan)

Topotecan and Irinotecan are cytotoxic chemotherapies that inhibit the TOP1 enzyme. They disrupt normal replication and transcription processes to induce DNA damage and apoptosis in rapidly dividing cells. Resistance to TOP1 inhibition can occur as a result of mutations in TOP1 or in cells not undergoing DNA replication; whereas, hypersensitivity can arise due to deficiencies in checkpoint and DNA-repair pathways [21].

In the CCLE panel, these two TOP1 inhibitors showed largely similar pharmacological effects based on IC50 values (**Figure 2**). We applied PC-Meta to each drug dataset and identified 757 and 211 pan-cancer gene markers associated with response to Topotecan and Irinotecan respectively (**Table 1** **; Table S5**). The discordant number of markers identified for these two drugs may have resulted from differences in drug actions or the different number of cell lines screened for each drug – 480 for Topotecan and 303 for Irinotecan. Nonetheless, 134 out of the 211 (63.5%) gene markers identified for Irinotecan still overlapped with those identified for Topotecan and are likely associated with general mechanisms of TOP1 inhibition (**Table 1**).

**Figure 3:**
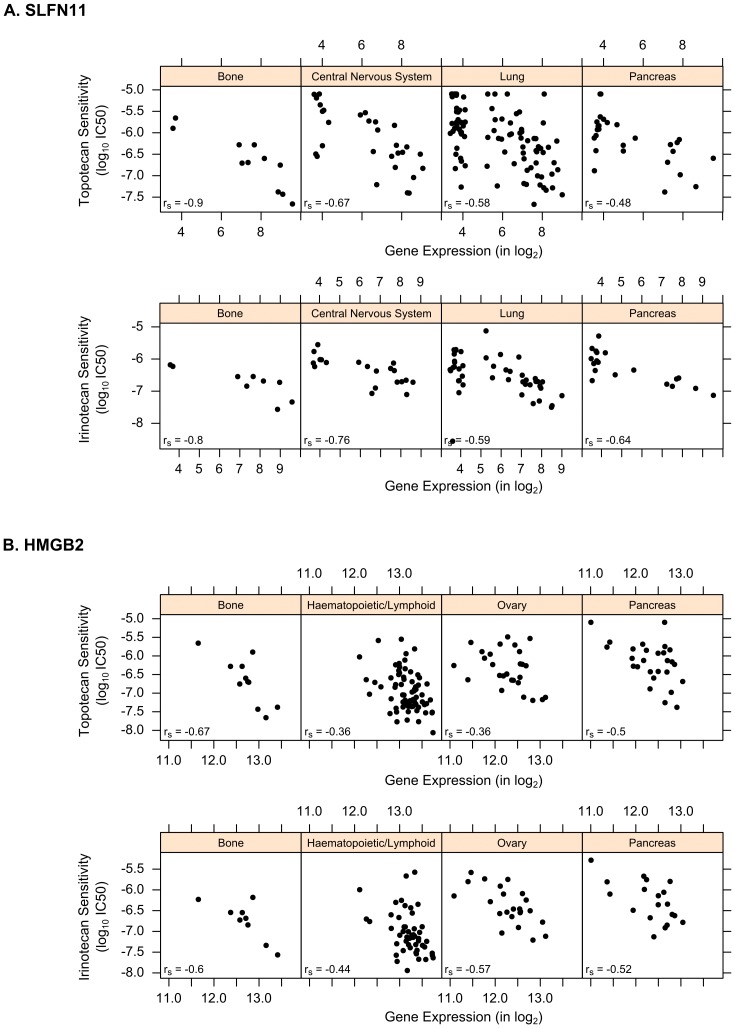
Top markers of response to TOP1 inhibitors: (A) SLFN11 and (B) HMGB2. Scatter plots show correlation between gene expression and pharmacological response values across several cancer lineages, where up-regulation of SLFN11 and HMGB2 correlate with drug sensitivity (indicated by smaller IC50 values).

Out of the 134 common genes identified for the two drugs by PC-Meta (**Table S3**), many are highly correlated with response (based on meta-FDR values) and have known functions that can affect the cytotoxicity of TOP1 inhibitors. For example, the top gene marker Schlafen family member 11 (SLFN11) showed increased expression in cell lines sensitive to both Topotecan and Irinotecan across ten individual cancer lineages (**Figure 3A**). This significant trend (meta-FDR  = 6.4×10^−18^ for Topotecan and 1.9×10^−10^ for Irinotecan; see Methods) agrees with recent studies delineating SLFN11's role in sensitizing cancer cells to DNA-damaging agents by enforcing cell cycle arrest and induction of apoptosis [8], [22]. Another top marker, high-mobility group box 2 (HMGB2), is a mediator of genotoxic stress response and showed reduced expression in cell lines resistant to TOP1 inhibitors in multiple lineages (**Figure 3B**; meta-FDR  = 1.7×10^−07^ for Topotecan and 3.7×10^−03^ for Irinotecan). This coincides with previous findings showing that abrogated HMGB2 expression results in resistance to chemotherapy-induced DNA damage [23]. Similarly, BCL2-Associated Transcription Factor 1 (BCLAF1), a regulator of apoptosis and double-stranded DNA repair, was also down-regulated in drug-resistant cell lines (meta-FDR  = 4.8×10^−04^ for Topotecan and 1.9×10^−03^ for Irinotecan), which is concordant with its previously observed suppression in intrinsically radioresistant cell lines [24].

**Figure 4:**
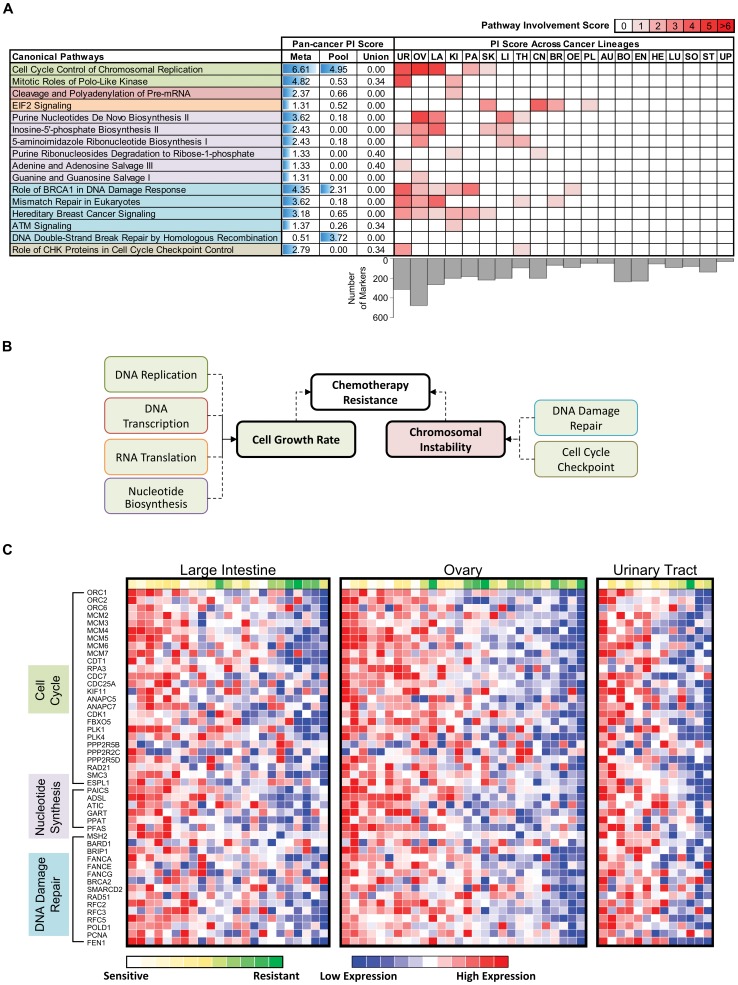
Pan-cancer analysis of TOP1 inhibitor Topotecan. (A) Pan-cancer pathways with significant involvement in drug response detected by PC-Meta, PC-Pool, PC-Union approaches (on the left). These pathways can be grouped into six biological processes (distinguished by background color), which converge on two distinct mechanisms. The involvement level of these pan-cancer pathways predicted by different approaches is illustrated with blue horizontal bars. Pathway involvement in each cancer lineage predicted by PC-Meta is indicated by the intensity of red fills in corresponding table (on the right). Pan-cancer and lineage-specific pathway involvement (PI) scores are derived from pathway enrichment analysis and calculated as -log_10_(BH-adjusted p-values). Only the top pathways with PI scores >1.3 are shown. Cancer lineage abbreviations – AU: autonomic; BO: bone; BR: breast; CN: central nervous system; EN: endometrial; HE: haematopoetic/lymphoid; KI: kidney; LA: large intestine; LI: liver; LU: lung; OE: oesophagus; OV: ovary; PA: pancreas; PL: pleura; SK: skin; SO: soft tissue; ST: stomach; TH: thyroid; UP: upper digestive; UR: urinary (B) Predicted known and novel mechanisms of intrinsic response to TOP1 inhibition. Red- and green-fill indicate increased and decreased activity in drug-resistant cell-lines respectively. (C) Heatmap showing the expression of genes in the cell cycle, nucleotide synthesis, and DNA damage repair pathways correlated with Topotecan response in multiple cancer lineages.

To investigate pan-cancer mechanisms underlying variations in Topotecan response, we mapped the entire set of pan-cancer gene markers identified by PC-Meta onto corresponding cell signaling pathways (using IPA pathway enrichment analysis). Each pathway was assigned a ‘pathway involvement (PI) score’ defined as –log_10_ of the pathway enrichment p-value, and pathways with PI scores > = 1 were considered to have significant influence on response. On the Topotecan dataset, PC-Meta detected 15 pan-cancer pathways relevant to drug response (PI scores  = 1.3–6.6), with the most significant pathways related to cell cycle regulation and DNA damage repair (**Figure 4A** **;** **Table 2**). In contrast, the same enrichment analysis yielded only 3 significantly enriched pathways for PC-Pool markers and no significant pathways for PC-Union markers. Clearly, the identification of more significant pathways by PC-Meta can be attributed to the increased power of our approach to pinpoint additional potentially relevant gene markers compared to PC-Pool and PC-Union (757 vs. 474 and 61 respectively; **Table 1**).

**Table 2:** Component genes of top pan-cancer pathways associated with drug response.

**Topotecan**	
Cell Cycle Control of Chromosomal Replication	ORC1(9), MCM6(6), ORC2(6), CDT1(4), MCM2(4), MCM4(4), RPA3(4), MCM5(3), MCM7(3), ORC6(3), CDC7(2), MCM3(2)
Mitotic Roles of Polo-Like Kinase	KIF11(6), ANAPC5(5), ANAPC7(5), CDK1(5), FBXO5(4), CDC25A(3), PLK4(3), PPP2R5D(3), RAD21(3), SMC3(3), CDC7(2), PLK1(2), PPP2R5B(2), ESPL1(1), PPP2R2C(1)
Cleavage and Polyadenylation of Pre-mRNA	CPSF2(5), NUDT21(5), PAPOLA(5), CPSF6(3), CSTF3(3)
EIF2 Signaling	RPL4(7), EIF3H(6), RPL36(6), **EIF2AK3**(5), EIF3A(5), EIF3D(5), EIF3E(5), PPP1CC(5), RPL11(5), **AGO2**(4), EIF2S1(4), EIF3L(4), RPL5(4), RPL8(4), RPLP2(4), RPS6(4)
Purine Nucleotides De Novo Biosynthesis II	PAICS(6), ADSL(5), ATIC(5), GART(5), PPAT(5), PFAS(3)
Adenine and Adenosine Salvage III	HPRT1(4), PNP(4), ADAT3(3)
Role of BRCA1 in DNA Damage Response	MSH2(7), FANCA(6), RFC5(6), BARD1(5), BRIP1(5), FANCG(5), BRCA2(4), SMARCD2(4), FANCE(3), RAD51(3), RFC2(3), RFC3(3), PLK1(2)
Mismatch Repair in Eukaryotes	MSH2(7), RFC5(6), POLD1(5), PCNA(4), FEN1(3), RFC2(3), RFC3(3)
ATM Signaling	CDK1(5), TDP1(5), MAPK8(4), SMC2(4), CDC25A(3), CREB1(3), RAD51(3), SMC3(2)
DNA Double-Strand Break Repair by Homologous Recombination	BRCA2(4), LIG1(4), RAD51(3)
Hereditary Breast Cancer Signaling	MSH2(7), FANCA(6), POLR2D(6), POLR2F(6), RFC5(6), BARD1(5), CDK1(5), FANCG(5), **HDAC11**(5), SMARCD2(5), BRCA2(4), FANCE(3), POLR2I(3), RAD51(3), RFC2(3), RFC3(3)
Role of CHK Proteins in Cell Cycle Checkpoint Control	RFC5(6), CDK1(5), CLSPN(4), PCNA(4), CDC25A(3), PPP2R5D(3), RFC2(3), RFC3(3), PLK1(2), **PPP2R5B**(1)
**Panobinostat**	
Interferon Signaling	IFIT3(8), IRF1(6), IFIT1(5), IFITM1(5), IRF9(4), PSMB8(4), RELA(4), STAT2(4), TAP1(3)
Hepatic Fibrosis/Hepatic Stellate Cell Activation	FGF2(7), TGFBR2(7), EGFR(6), IL6(6), TIMP1(6), CCL2(5), CCL5(5), IGFBP3(5), MYH9(5), SMAD3(5), VEGFA(5), IL1B(4), RELA(4), TIMP2(4), FGF1(3), IL8(3), MMP1(3), TGFB2(3)
Glucocorticoid Receptor Signaling	SMARCD2(7), TGFBR2(7), IL6(6), NR3C1(6), POU2F1(6), ADRB2(5), CCL2(5), CCL5(5), EP300(5), RRAS2(5), SMAD3(5), HMGB1(4), IL1B(4), MAP3K14(4), PIK3C2B(4), POLR2F(4), RELA(4), TAF3(4), IL8(3), MMP1(3), SERPINE1(3), SLPI(3), TGFB2(3), HLTF(2)
Antigen Presentation Pathway	HLA-C(5), TAP2(5), PSMB8(4), PSMB9(4), TAP1(3)
NF-κB Signaling	TGFBR2(7), EGFR(6), UBE2N(6), EP300(5), FGFR4(5), RRAS2(5), IL1B(4), MAP3K14(4), PIK3C2B(4), RELA(4), TNIP1(4), EIF2AK2(3), NGF(3)
Granzyme A Signaling	ANP32A(6), EP300(5), HIST1H1E(5), NME1(5)
Caveolar-mediated Endocytosis Signaling	EGFR(6), FLNA(6), CAV1(5), HLA-C(5), ITGA5(5), PTRF(4)
**PD-0325901**	
Human Embryonic Stem Cell Pluripotency	BDNF(8), NGF(6), FZD2(5), MRAS(5), S1PR1(5), TGFB2(5), FGF2(3)
Neurotrophin/TRK Signaling	BDNF(8), SPRY2(7), NGF(6), MRAS(5)

Note: Number in parentheses indicates the number of cancer lineages that each gene was predicted to be involved in. Genes in regular and bolded font are down- and up-regulated in resistant cell lines respectively. For pathways with many overlapping component genes, the best representative pathway is listed. Full list of pathways is available in **Table S6**.

The pathways detected by PC-Meta converged onto two major mechanisms that could influence chemotherapy response: cellular growth rate and chromosomal instability (**Figure 4A–B**). All genes involved in cell cycle control, DNA transcription, RNA translation, and nucleotide synthesis processes were down-regulated in chemotherapy-resistant cell lines, which suggested slower growth kinetics as a mechanism of resistance. Most genes involved in DNA damage repair and cell cycle checkpoint regulation were also down-regulated in resistant cell lines. This may appear counterintuitive because repair pathways typically mitigate DNA damage-induced cell death (as caused by TOP1 inhibitors). However, some of their component genes (such as RAD51, BRCA2, and FANC-family genes) are also key regulators of genomic stability and their disruption can reflect a genome instability phenotype that is inherently resistant to genotoxic stress from chemotherapy [25], [26]. In fact, our finding agrees with a recently reported DNA repair gene signature that was predictive of both homologous repair suppression contributing to genome instability as well as sensitivity to chemotherapy in patient studies [27]. Enrichment analysis performed on the Irinotecan marker set revealed similar dysregulated pathways related to cell cycle control and DNA damage repair (**Table S6**). This suggests these two mechanisms are generally important for managing TOP1 inhibition.

Since recurrent drug response pathways may be involved in only a subset of cancer types, we aimed to delineate the extent of their roles in each cancer lineage. A subset of pan-cancer markers significantly correlated with response in each cancer type were selected as ‘lineage-specific markers’. Then, each set of lineage-specific markers was assessed for enrichment to calculate a PI score for each pan-cancer pathway in each lineage. Interestingly, the pan-cancer pathways relevant to Topotecan response exhibited obvious lineage-specific differences (**Figure 4A**). Intrinsic response in urinary, ovarian and large intestine cancers appeared prominently influenced through multiple mechanisms including cell cycle regulation, nucleotide synthesis, and DNA repair pathways (**Figure 4C**), whereas response in central nervous system cancers primarily involved EIF2 signaling. One-third of the cancer lineages were not characterized by any pan-cancer response mechanisms. Lineages without significant PI scores generally had fewer detected lineage-specific markers (**Figure 4A**), but not in all cases – bone and endometrial cancers had a similar number of markers to urinary and large intestine cancers, two lineages with the most significant PI scores.

### Intrinsic Determinants of Response to HDAC Inhibitor (Panobinostat)

Panobinostat (LBH-589) is a pan-histone deacetylase (HDAC) inhibitor, which causes the hyperacetylation of histone and non-histone proteins. This triggers a plurality of anti-cancer mechanisms through both transcriptional and post-translational processes, including the activation of apoptotic pathways and the degradation of oncogenic HSP90 client proteins [28]. Resistance to HDAC inhibition has been associated with numerous mechanisms including enforced expression of anti-apoptotic proteins, activation of MAPK/PI3K/STAT3 signaling pathways, and the activation of NFkB pathway [28].

**Figure 5:**
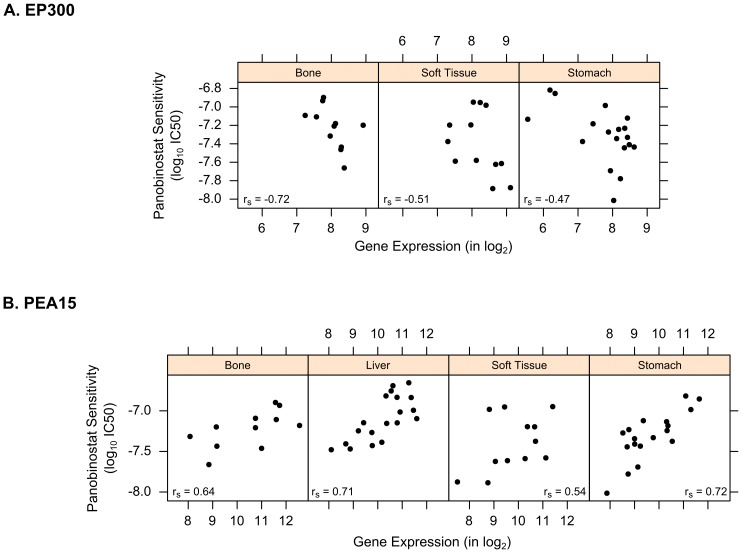
Top gene markers of response to HDAC inhibitor Panobinostat: (A) EP300 and (B) PEA15. Scatter plots show correlation between gene expression and pharmacological response values across several cancer lineages, where down-regulation of EP300 and up-regulation of PEA15 correlate with drug resistance (indicated by greater IC50 values).

Application of the PC-Meta analysis identified 542 pan-cancer gene markers associated with intrinsic response to Panobinostat (**Table 1** **; Table S5**). One of the top markers identified by PC-Meta was the histone acetyltransferase (HAT) enzyme EP300, which antagonizes HDACs. It had reduced expression in drug-resistant cell lines across five cancer lineages (Figure 5A; meta-FDR = 8.9×10-3). In previous studies, lower EP300 expression has been shown to boost HDAC influence and attenuate the effects of HDAC inhibition [28]. Another interesting top pan-cancer gene marker, PEA-15, has anti-apoptotic function and was up-regulated in the resistant cell lines of seven cancer lineages (Figure 5B; meta-FDR = 2.7×10-5). Since PEA-15 overexpression can suppress FAS/TNFα-mediated cell death, it may counteract the effects of HDAC inhibitors on the extrinsic apoptotic pathway [28], [29].

**Figure 6:**
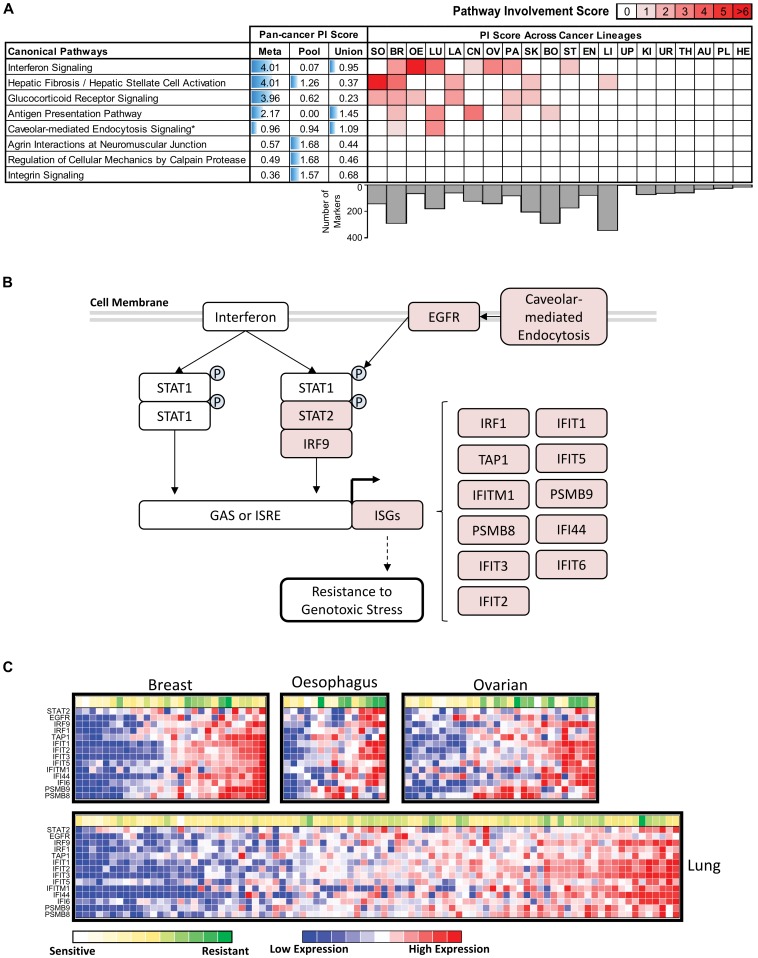
Pan-cancer analysis of HDAC inhibitor Panobinostat. (A) Pan-cancer pathways with significant involvement in drug response detected by PC-Meta, PC-Pool, PC-Union approaches (on the left). The predicted involvement level of these pan-cancer pathways by different approaches is illustrated with blue horizontal bars (in the middle). The involvement of these pan-cancer pathways in each cancer lineage predicted by PC-Meta is indicated by the intensity of red fills in corresponding table (on the right). Pan-cancer and lineage-specific pathway involvement (PI) scores are derived from pathway enrichment analysis and calculated as -log_10_(BH-adjusted p-values). Only the top pathways with PI scores >1.3 are shown. Cancer lineage abbreviations – AU: autonomic; BO: bone; BR: breast; CN: central nervous system; EN: endometrial; HE: haematopoetic/lymphoid; KI: kidney; LA: large intestine; LI: liver; LU: lung; OE: oesophagus; OV: ovary; PA: pancreas; PL: pleura; SK: skin; SO: soft tissue; ST: stomach; TH: thyroid; UP: upper digestive; UR: urinary (B) The predicted role of STAT/Interferon signaling pathway in Panobinostat inhibition. Red- and green-fills indicates increased and decreased gene expression in drug-resistant cell-lines respectively. (C) Heatmap showing the expression of genes in the STAT/Interferon pathway correlated with Panobinostat response in multiple cancer lineages.

To investigate pan-cancer mechanisms of response to Panobinostat, we applied pathway enrichment analysis to the set of PC-Meta pan-cancer gene markers. This revealed 20 pathways significantly associated with response with PI scores ranging from 1.0 to 4.0 (**Figure 6A** **;** **Table 2**). In contrast, enrichment analysis based on gene markers derived from PC-Pool and PC-Union identified only 6 and 8 pathways respectively, even though the PC-Pool approach provided greater number of gene markers than PC-Meta (723 vs 542). The PI scores for commonly detected pathways (e.g. Hepatic Stellate Cell Activation) were significantly higher for gene markers derived by PC-Meta compared to the two alternative pan-cancer analysis methods. Similar to our conclusions for the TOP1 inhibitors, PC-Meta performed better than alternative approaches in identifying pathways potentially involved in response to Panobinostat.

The pan-cancer pathways predicted by PC-Meta to be most associated with response were Interferon Signaling, Glucocorticoid Receptor (GR) Signaling, and Hepatic Stellate Cell (HSC) Activation (**Figure 6A**). Transient overexpression of the Interferon signalling pathway has been shown to trigger anti-viral/anti-pathogen immune responses as well as inhibit cell proliferation and induce apoptosis. However, recent studies showed that the constitutive overexpression of Interferon signaling confers resistance to genotoxic stress/damage possibly due to inability of a cell to transmit cytotoxic response signals [30], [31]. The latter was in line with our observations that genes in this pathway, such as interferon-stimulated genes (ISG), were overexpressed in drug-resistant cell lines across seven cancer lineages (**Figure 6B–C**). Interestingly, we also observed that the caveolar-mediated endocytosis signaling pathway had significant involvement in response specifically in lung cancers. Caveolar trafficking pathways can internalize various membrane receptors such as EGFR, and thereby strengthen EGFR signaling [32] and downstream activation of Interferon/STAT-1 signaling. Therefore, we speculate that the collective overexpression of caveolar-mediated endocytosis, EGFR, and Interferon/STAT-1 signaling pathway genes can coordinate stronger inherent resistance to Panobinostat in a subset of lung cancers.

GR signaling pathway, the second most enriched pathway in our analysis, is a regulator of immune responses as well as cellular apoptosis and proliferation. It comprises a number of genes that were overexpressed in the drug-resistant cell lines across several cancer lineages (**Table 2**), such as the nuclear hormone receptor GR/NR3C1 and RELA component of NF-kB complex. The expression of nuclear hormone receptor GR/NR3C1 normally drives the induction of anti-apoptotic proteins through the downstream activation of NF-kB signaling; however, this function can be compromised in absence of HDAC6 [33]. Therefore, we speculate that the observed up-regulations of GR/NR3C1 and NF-kB can oppose loss GR function resulting from HDAC inhibition [34]. Several genes with anti-apoptotic functions comprising the HSC Activation pathway, the third most enriched pathway, also had up-regulated expression in drug-resistant cell lines. These included members of the tissue inhibitor of metalloproteinase family (TIMP1 and TIMP2) that mediate cell survival [35], members of the fibroblast growth factor family (FGF1, FGF2) that up-regulate anti-apoptosis proteins and have broad cytoprotective effects across cancer types, and member of the vascular endothelial growth factor (VEGF1) that has also demonstrated pro-survival effects [36]. Collectively, these findings suggest that the up-regulation of cell survival through a complex diversity of molecular regulators is likely to be a primary modulator of response to Panobinostat across diverse cancer lineages.

### Intrinsic Determinants of Response to MEK Inhibitors (PD-0325901 and AZD6244/Selumetinib)

MEK inhibitors have shown promise in treating cancers addicted to oncogenic mutations that dysregulate the RAF/MEK/ERK signaling pathway. For example, activating BRAF mutations occur in roughly 7% of all cancers, including up to 70% of melanomas, 22% of colorectal cancers, and 30% of serous ovarian cancers, and can confer sensitivity to MEK inhibition [37]. Resistance to MEK inhibition can occur as a result of molecular alterations upstream in the RAF/MEK/ERK pathway (e.g. KRAS amplifications or EGFR mutations) as well as activating mutations in the PI3K/AKT/MTOR pathway, which regulates similar mechanisms in apoptosis and cell growth [38].

**Figure 7:**
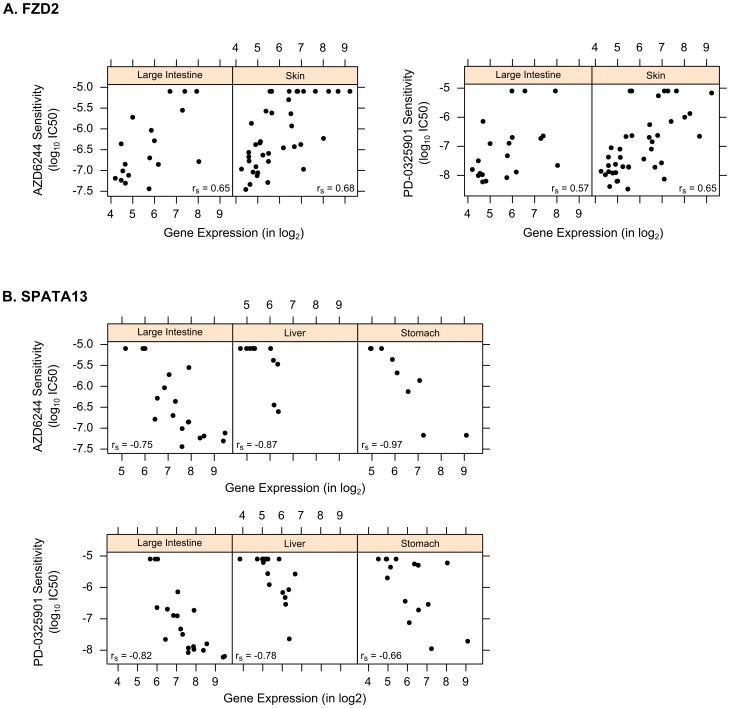
Top gene markers of response to MEK inhibitors PD-0325901 and AZD6244: (A) FZD2 and (B) SPATA13. Scatter plots show correlation between gene expression and pharmacological response values across several cancer lineages, where up-regulation of FZD2 and down-regulation of SPATA13 correlate with drug resistance (indicated by greater IC50 values).

**Figure 8:**
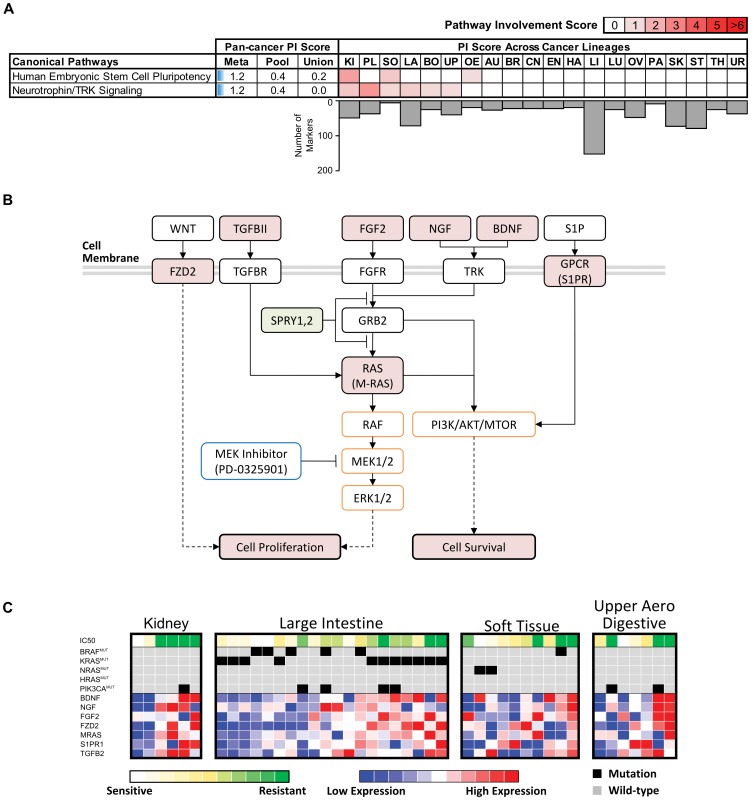
Pan-cancer analysis of MEK Inhibitor PD-0325901. (A) Pan-cancer pathways with significant involvement in drug response detected by PC-Meta, PC-Pool, PC-Union approaches (on the left). The predicted involvement level of these pan-cancer pathways by different approaches is illustrated with blue horizontal bars (in the middle). The involvement of these pan-cancer pathways in each cancer lineage predicted by PC-Meta is indicated by the intensity of red fills in corresponding table (on the right). Pan-cancer and lineage-specific pathway involvement (PI) scores are derived from pathway enrichment analysis and calculated as -log_10_(BH-adjusted p-values). Cancer lineage abbreviations – AU: autonomic; BO: bone; BR: breast; CN: central nervous system; EN: endometrial; HE: haematopoetic/lymphoid; KI: kidney; LA: large intestine; LI: liver; LU: lung; OE: oesophagus; OV: ovary; PA: pancreas; PL: pleura; SK: skin; SO: soft tissue; ST: stomach; TH: thyroid; UP: upper digestive; UR: urinary (B) The predicted role of PC-Meta identified compensatory mechanisms in MEK inhibition. Red- and green-fills indicates increased and decreased gene expression or activity in drug-resistant cell-lines respectively. Downstream RAF/MEK/ERK and PI3K/AKT/MTOR pathways are indicated in orange boxes and inhibitor is indicated in blue box. (C) Heatmap showing the expression of genes in the PC-Meta detected compensatory pathways correlated with PD-0325901 resistance in multiple cancer lineages.

We investigated two experimental MEK inhibitors currently undergoing clinical trials: PD-0325901 and AZD6244 (Selumetinib). Both drugs showed similar patterns of pharmacological sensitivity across the panel of cancer lineages (**Figure 2**). However, these drugs and their response data are characterized by important differences: PD-0325901 is 10-times more potent than AZD6244 as a MEK inhibitor [39] and these drugs were screened on different numbers of cell lines (PD-0325901 on 366 and AZD6244 on 247). Our PC-Meta analysis yielded 171 response markers for the more potent PD-0325901 and only 10 response markers for AZD6244 (**Table S5**). Although this high discrepancy was unexpected, we believe it can be partly attributed to the aforementioned differences. Nevertheless, 8/10 (80%) of the AZD6244 gene markers were shared with PD-0325901 and may represent promising markers of resistance to the family of MEK inhibitors (**Table S4**). In particular, three of the identified genes were previously published as a part of the MEK-response gene signature [12]. These included SPRY2 that was down-regulated in resistant cell lines (meta-FDR  = 1.4×10^−3^ for PD-0325901 and 4.0×10^−3^ for AZD6244), FZD2 that was up-regulated (**Figure 7A**; meta-FDR  = 1.5×10^−4^ for PD-0325901 and 6.0×10^−3^ for AZD6244) and CRIM1 (meta-FDR  = 1.6×10^−5^ for PD-0325901 and 5.0×10^−3^ for AZD6244) that was also up-regulated in resistant cells, consistent with previous findings (**Figure 8**). The observed decrease in expression of other common genes such as SPATA13 (**Figure 7B**), LYZ, and MGST2, to our knowledge, have not yet been implicated in resistance to MEK inhibitors and thus invites further investigation.

We selected the more potent and broadly screened PD-0325901 to further characterize mechanisms of intrinsic response to MEK inhibition. Pathway enrichment analysis of the PC-Meta pan-cancer gene markers resulted in only two significant pathways (**Figure 8A** **;** **Table 2**). Strikingly, no significant pathways were detected from PC-Pool or PC-Union gene markers. This result may be partially attributed to the limited number of markers for PC-Pool (46), but not for PC-Union (156), which detected a comparable number of genes as PC-Meta (**Table 1**).

The two pathways discovered by PC-Meta, Neutrophin/TRK signaling and Human Embryonic Stem Cell Pluripotency comprise numerous genes located upstream of the MEK target whose dysregulations can activate the PI3K signaling pathway and drive resistance to MEK inhibition. (**Figure 8B**). The neutrophin growth factors NGF and BDNF and the fibroblast growth factor FGF2 can trigger PI3K signaling through RAS and adaptor protein GRB2 [40]. These growth factors were overexpressed in PD-0325901-resistant cell lines. Additionally, the relevance of FGF2 regulated signaling appears to be reinforced through the suppressed expression of FGF antagonists SPRY1/2 in drug-resistant cell lines [36]. Interestingly, M-RAS, a close relative of classical RAS proteins (e.g. K-RAS, N-RAS), can also activate downstream PI3K/AKT effectors [41], and had elevated expression in resistant cell lines. Finally, in resistant cell lines, we observed up-regulation of gamma-protein coupled receptor S1PR, which can also stimulate the PI3K/AKT pathways [42] as well as the up-regulation of transforming growth factor beta TGFBII, which has been recently implicated in resistance to MEK-inhibitor AZD6244 [43]. Altogether, our findings support existing knowledge of PI3K pathway involvement as a principal mechanism of resistance to MEK inhibitors. Additionally, the seven genes identified through our analysis may serve as a useful gene signature of such resistance.

Since mutations in the RAS/MEK/ERK or PI3K/AKT/MTOR pathways have been linked to the response to MEK inhibitors, we evaluated these mutations against our seven-gene signature in predicting drug response (**Figure 8C**). The mean expression of the seven-gene resistance signature was significantly correlated with response values in three cancer lineages: kidney cancers (Spearman's rho  = 0.85, p-value  = 0.017), large intestine/colorectal cancers (Spearman's rho  = 0.61, p-value  = 0.002), and soft tissue cancers (Spearman's rho  = 0.61, p-value  = 0.031). In contrast, individual mutation events were significantly associated with response in fewer cancer lineages. For instance, BRAF mutations were associated with drug response values in only large intestinal/colorectal cancers (Student's t-test, p-value  = 0.024). Of the multiple RAS proteins (KRAS, NRAS, HRAS) whose mutation are known to drive oncogenic MEK pathway activation [44], [45], only NRAS mutations were associated with drug response values in soft tissue cancers (Student's t-test, p-value  = 0.003). Finally, PIK3CA mutations, which can confer inappropriate activation of the PI3K signaling pathway, were weakly associated with drug-resistance in cancers of the large intestine and upper aerodigestive tract (Student's t-test, p-value  = 0.003 in both). Altogether, these findings underscore the fact that known mutations cannot fully explain the response in entire cancer population. Importantly, it illustrates the advantages of our PC-Meta approach to identify potentially important compensatory mechanisms by which cancers resist targeted therapies.

## Conclusions

In this study, we investigated the inherent determinants of cancer drug response across multiple cancer lineages. For this purpose, we developed a pan-cancer analysis strategy based on meta-analysis, PC-Meta, and comprehensively characterized known and novel mechanisms of response to both cytotoxic chemotherapies and targeted therapies in the publically available CCLE resource. Since many CCLE compounds were not amenable to comprehensive analysis due to highly biased pharmacological profiles or lack of reasonable sample sizes, we focused on a subset of five drugs that exhibited a broad range of *in vitro* sensitivity values across numerous cancer lineages. Importantly, compared to alternative approaches, our PC-Meta approach consistently demonstrated higher power in identifying potentially relevant markers and ability to infer the mechanisms of response.

For TOP1 inhibitors that are dependent on DNA replication and transcription rates, our analysis predicted cell lines with slower growth kinetics as inherently more drug-resistant irrespective of cancer lineage. Although this was not unexpected, our predictions suggested that the cellular growth rates in different cancer types can be suppressed through down-regulation of several processes including cell cycle control, nucleotide synthesis, and RNA translation. The degree of involvement of specific pathways in each cancer lineage can guide selection of proper combination therapy to circumvent resistance. We further observed that the overexpression of DNA repair genes may be indicative of a genome instability phenotype that may confer intrinsic resistance to TOP1 inhibition.

For Panobinostat, a pan-HDAC inhibitor that has been hypothesized to act on cancer cells through a number of diverse mechanisms, we identified the up-regulation of STAT-1/interferon signaling as a principal factor of inherent resistance across multiple cancer lineages. The basal overexpression of this pathway has been previously implicated in resistance to both radiotherapy and chemotherapy in lung and breast cancers, where it was suggested to confer resistance to genotoxic stress and damage as a result of failing to transmit cytotoxic signals. Our results expand its importance for additional cancer types such as those arising from ovarian and oesophageal tissue. Interestingly, our approach also identified a set of lung-specific markers involved in the caveolar-mediated endocytosis signaling, suggesting an important role of this pathway in the resistance of lung cancers to Panobinostat.

For MEK inhibitors, our PC-Meta analysis identified multiple determinants of inherent resistance that are upstream of the targeted MEK. These determinants include up-regulation of alternative oncogenic growth factor signaling pathways (e.g. FGF, NGF/BDNF, TGF) in resistant cell lines. In particular, we speculate that the up-regulation of the neutrophin-TRK signaling pathway can induce resistance to MEK-inhibition through the compensatory PI3K/AKT pathway and may serve as a promising new marker. We also identified the overexpression of MRAS, a less studied member of the RAS family, as a new indicator of drug-resistance. Importantly, our analysis demonstrated that gene expression markers identified by PC-Meta provides greater power in predicting *in vitro* pharmacological sensitivity than known mutations (such as in BRAF and RAS-family proteins) that are known to influence response. This emphasizes the importance of continuing efforts to develop gene expression based markers and warrants their further evaluation on multiple independent datasets.

In conclusion, we have developed a meta-analysis approach for identifying inherent determinants of response to chemotherapy. Our approach avoids the significant loss of signal that can potentially result from using the standard pan-cancer analysis approach of directly pooling incomparable pharmacological and molecular profiling data from different cancer types. Application of this approach to three distinct classes of inhibitors (TOP1, HDAC, and MEK inhibitors) available from the public CCLE resource revealed recurrent markers and mechanisms of response, which were supported by findings in the literature. This study provides compelling leads that may serve as a useful foundation for future studies into resistance to commonly-used and novel cancer drugs and the development of strategies to overcome it. We make the compendium of markers identified in this study available to the research community.

## Supporting Information

Figure S1 **Drug response across different lineages for 24 CCLE compounds.** Boxplots indicate the distribution of drug sensitivity values (based on IC50) in each cancer lineage for each cancer drug. For example, most cancer lineages are resistant to L-685458 (IC50 around 10^−5^ M) except for haematopoietic cancers (IC50 from 10^−5^ to 10^−8^ M). The number of samples in a cancer lineage screened for drug response is indicated under its boxplot. Cancer lineage abbreviations – **AU**: autonomic; **BO**: bone; **BR**: breast; **CN**: central nervous system; **EN**: endometrial; **HE**: haematopoetic/lymphoid; **KI**: kidney; **LA**: large intestine; **LI**: liver; **LU**: lung; **OE**: oesophagus; **OV**: ovary; **PA**: pancreas; **PL**: pleura; **SK**: skin; **SO**: soft tissue; **ST**: stomach; **TH**: thyroid; **UP**: upper digestive; **UR**: urinary.
(TIF)

Table S1 **Summary of PC-Meta, PC-Pool, and PC-Union markers identified for all CCLE drugs (meta-FDR <0.01).**
(XLSX)

Table S2 **Functions significantly enriched in the PC-Pool gene markers associated with sensitivity to L-685458.**
(XLS)

Table S3 **Overlap of PC-Meta markers between TOP1 inhibitors, Topotecan and Irinotecan.**
(XLSX)

Table S4 **Overlap of PC-Meta markers between MEK inhibitors, PD-0325901 and AZD6244, and reported signature in** [12] **.**
(XLSX)

Table S5 **List of significant PC-Meta pan-cancer markers identified for each of 20 drugs.**
(XLSX)

Table S6 **Pan-cancer pathways with predicted involvement in response to TOP1, HDAC, and MEK inhibitors.**
(XLSX)
