# Supplementary figures and images for: A Meta-Analysis Approach for Characterizing Pan-Cancer Mechanisms of Drug Sensitivity in Cell Lines
Source: PLoS One. 2014 Jul 18;9(7):e103050. doi: 10.1371/journal.pone.0103050 (PMC4103868; doi:10.1371/journal.pone.0103050)

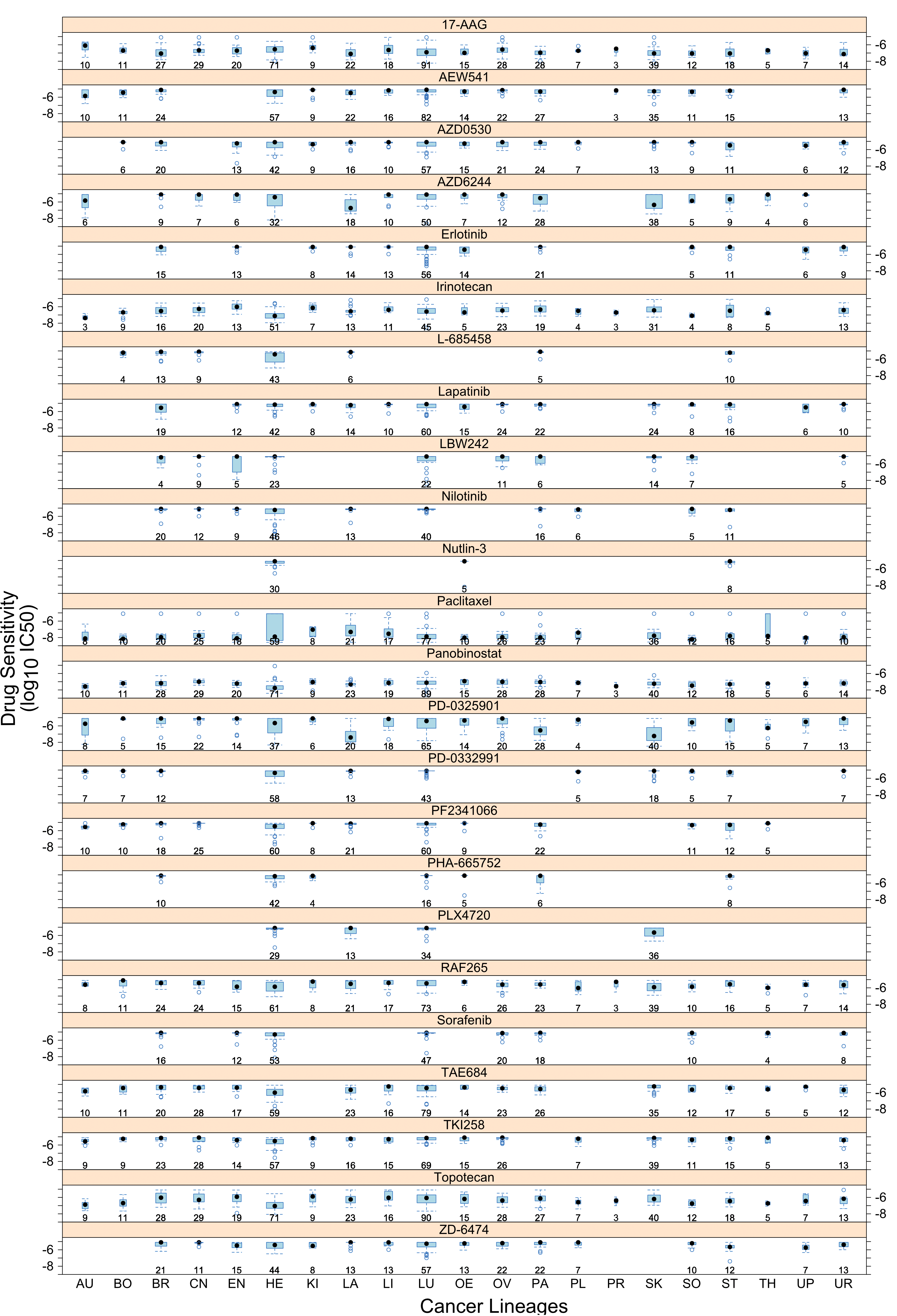

Supplement: Figure S1 — Drug response across different lineages for 24 CCLE compounds. Boxplots indicate the distribution of drug sensitivity values (based on IC50) in each cancer lineage for each cancer drug. For example, most cancer lineages are resistant to L-685458 (IC50 around 10−5 M) except for haematopoietic cancers (IC50 from 10−5 to 10−8 M). The number of samples in a cancer lineage screened for drug response is indicated under its boxplot. Cancer lineage abbreviations – AU: autonomic; BO: bone; BR: breast; CN: central nervous system; EN: endometrial; HE: haematopoetic/lymphoid; KI: kidney; LA: large intestine; LI: liver; LU: lung; OE: oesophagus; OV: ovary; PA: pancreas; PL: pleura; SK: skin; SO: soft tissue; ST: stomach; TH: thyroid; UP: upper digestive; UR: urinary. (TIF) [file pone.0103050.s001.tif]
